# Cultural and behavioral drivers of zoonotic disease transmission and persistence among diverse pastoralist communities in East Africa

**DOI:** 10.21203/rs.3.rs-5842512/v1

**Published:** 2025-04-17

**Authors:** Dismas CO. Oketch, Ruth Njoroge, Tonny O. Ngage, Asha Abdikadir Omar, Abdulai Magarre, Raphael Pasha, John Gachohi, Samuel Waiguru Muriuki, Samoel Ashimosi Khamadi, Ali Duba Boru, Boku Bodha, Lydia Kilowua, Nazaria Wanja Nyaga, Humphrey Kariuki Njaanake, Eunice Kamaara, Walter Jaoko, M. Kariuki Njenga, Eric Osoro, Dalmas Omia

**Affiliations:** Washington State University Global Health-Kenya; Washington State University Global Health-Kenya; University of Nairobi, Department of Anthropology, Gender and African Studies; University of Nairobi, Department of Anthropology, Gender and African Studies; Washington State University Global Health-Kenya; Washington State University Global Health-Kenya; Jomo Kenyatta University of Agriculture and Technology; Washington State University Global Health-Kenya; Kenya Medical Research Institute, Centre for Virus Research; County Government of Marsabit, Department of Health Services; County Government of Marsabit, Department of Veterinary Services; County Government of Kajiado, Department of Health; County Government of Kajiado, Department of Veterinary Services; University of Nairobi, Department of Medical Microbiology and Immunology; Moi University, School of Arts and Social Sciences; University of Nairobi, Department of Medical Microbiology and Immunology; Washington State University Global Health-Kenya; Washington State University Global Health-Kenya; University of Nairobi, Department of Medical Microbiology and Immunology

**Keywords:** One Health, Cultural, Pastoralist, Zoonotic, Transmission, Abortion, Brucella, Practices, Control

## Abstract

**Background:**

Zoonotic diseases such as brucellosis, Rift Valley fever, anthrax, rabies and bovine tuberculosis are highly prevalent among pastoralist communities in low-and middle-income countries.

**Methods:**

This study adopts a One Health approach, employing a range of participatory methods including informal observations, “go-along interviews,” narrative-, and key informant- interviews, to explore the cultural, behavioral, and structural drivers of zoonotic disease transmission among pastoralist communities in East Africa. We unpack how the physical environment, socio-economic systems, health systems, community influence and cultural competence as well as individual pastoralist’s unique characteristics, behaviors and lifestyles can be leveraged for effective public health interventions that reduce zoonotic risks and improve health outcomes for both humans and livestock.

**Results:**

We present data from 214 purposively selected participants, including 19 key informants, 68 in-depth interviews, 20 focus group discussions, and 22 direct ethnographic observations. Traditional knowledge and beliefs, risky cultural dietary practices such as consumption of raw milk, meat and blood, unprotected parturition assistance, unsafe disposal of carcasses and aborted fetuses were common and carried increased risk of zoonotic transmission. Women and children handled and milked small ruminants while adult men and *morans* (young warriors) were mostly involved with cattle, camels and slaughtering; hence exposing them to zoonotic pathogens disproportionately. There were piles of manure made up of animal excreta and secretions that were potentially highly contaminated with saprophytes and soil-borne zoonotic pathogens.

**Discussion:**

While livestock play a significant and indispensable role in the daily livelihoods of pastoralist communities, their close association of pastoralists with livestock coupled with their unique cultural and behavioral practices increases their risk of exposure to deadly zoonotic diseases. Although, most of these practices are environmentally and culturally adaptive, their risk for transmission is often overlooked. The study also highlights inadequate sanitary practices, poor disposal of animal carcasses and placentae and the absence of veterinary oversight in the production, distribution and consumption livestock products.

**Conclusions:**

Our study provides a holistic understanding of the subjective perspectives and nuanced insights underlying the emergence and persistence of zoonotic diseases within pastoralist communities. It also underscores the need for culturally sensitive One Health interventions that address these practices and enhance community awareness of zoonotic disease risks and prevention strategies which are often overlooked by conventional epidemiological studies.

## Introduction

Zoonotic diseases, such as brucellosis, Rift Valley fever, anthrax, rabies, and bovine tuberculosis, are prevalent among East African pastoralist communities, posing significant public health and socioeconomic challenges. These diseases impact both human health and livestock productivity, undermining livelihoods and food security in these low- and middle-income countries. Pastoralist communities engage in nomadic or semi-nomadic lifestyles, herding livestock across vast areas where animals from different households and locations co-mingle. This close human-livestock interaction, combined with cultural practices such as consuming raw animal products and reliance on ethnoveterinary knowledge, heightens zoonotic disease risks (Osoro et al., 2015; Ekwem et al., 2021). The traditional health-seeking behaviors and limited access to formal healthcare further exacerbate the challenges of disease control (Ole-Miaron, 2002; Kiringe, 2006; Meyer-Rochow, 2009; Sibeko, Johns and Cordeiro, 2021).

Brucellosis, the most common zoonotic disease globally, exemplifies these risks. With over 2.1 million new human cases reported annually(Laine et al., 2023), its prevalence in East Africa reaches as high as 46.5% in humans and 43.8% in livestock (Osoro et al., 2015; Njeru et al., 2016; Djangwani et al., 2021). Although livestock are the primary source of human infection, wild animals may act as reservoirs in regions with frequent human-wildlife interaction (Mick et al., 2014; Kamath et al., 2016).

In livestock, *Brucella spp.* are transmitted through direct contact with contaminated birth products such as placenta, aborted fetuses, fetal membranes, birth fluids and other tissues from other infected animals (González-Espinoza et al., 2021) or via indirect contact with contaminated environments and grazing fields (Corbel, 2006). *Brucella* spp. may also be transmitted by licking the genitalia of infected animals (El-Sayed and Awad, 2018), while infected males can transmit the infection to females through natural mating (Tulu, 2022). Human infection occurs primarily through ingestion of unpasteurized dairy products, direct contact with infected animals or animal tissues such as placenta, aborted fetuses, birth fluids and vaginal secretions, handling of sick animals, inhalation of the bacteria, and improper handling of animal carcasses (Corbel, 2006), underscoring the interconnected risks to human and animal health.

Despite extensive epidemiological research, there is limited understanding of how cultural and behavioral factors influence zoonotic disease transmission and control in pastoralist communities. Most studies emphasize quantitative health impacts (Njeru et al., 2016; Kemunto et al., 2018; Djangwani et al., 2021), neglecting the nuanced social and cultural dynamics that shape disease risks. Addressing this gap is essential for designing interventions that align with the unique practices and beliefs of these communities.

This study adopts a One Health approach, employing participatory methods to explore the cultural, behavioral, and structural drivers of zoonotic disease transmission among pastoralist communities in East Africa. By leveraging community perspectives, the research aims to inform culturally sensitive public health interventions that reduce zoonotic risks and improve health outcomes for both humans and livestock.

## Methods

### Study design

This was an ethnographic study in two pastoralist communities in East Africa – the Maasai and Rendile. The study employed various data collection techniques including go-along (walking) interviews, informal observations, individual in-depth interviews, focus group discussions and key informant interviews. Direct observations of the cultural practices around milking and milk consumption, parturition, handling of placentae, slaughtering and consumption of raw meat and blood as well as the interactions of humans, livestock and their shared environment were made.

Go-along interviews (Bergeron, Paquette and Poullaouec-Gonidec, 2014), and informal observations were held with local farmers, pastoralists, butchers and local herdsmen to help understand the use, cultural importance, and interactions of various biotic and abiotic factors within the ecosystem and how that influences the perspectives, beliefs and practices of the local community over the years. Go-along interviews and informal observations are innovative methods for capturing participants’ lived experiences in person, space and time. They were incorporated to aid in eliciting a deeper and more detailed understanding of the social processes, relations, behaviours, practices, perspectives, nuances, ambiguities, paradoxes and other contextual factors that other ethnographic methods may overlook. Go-along interviews involved the researchers go-along alongside a participant in their lived environment and asking questions along the way. This was particularly useful in gathering invaluable data about the lived experiences of *morans,* herders and other migrant groups.

Subsequently, individual and group interviews were conducted with purposively selected participants to construct detailed narratives on social, structural and cultural determinants and potential pathways of zoonotic disease transmission and control. Grounded theory (Brink et al., 2006) was used to capture pastoralists’ knowledge, beliefs, perceptions and practices.

### Study area and population

The study was conducted in Marsabit and Kajiado Counties. Both have diverse ecosystems, high density of livestock and rich cultural heritage relevant to zoonotic disease transmission.

**Figure 1:**
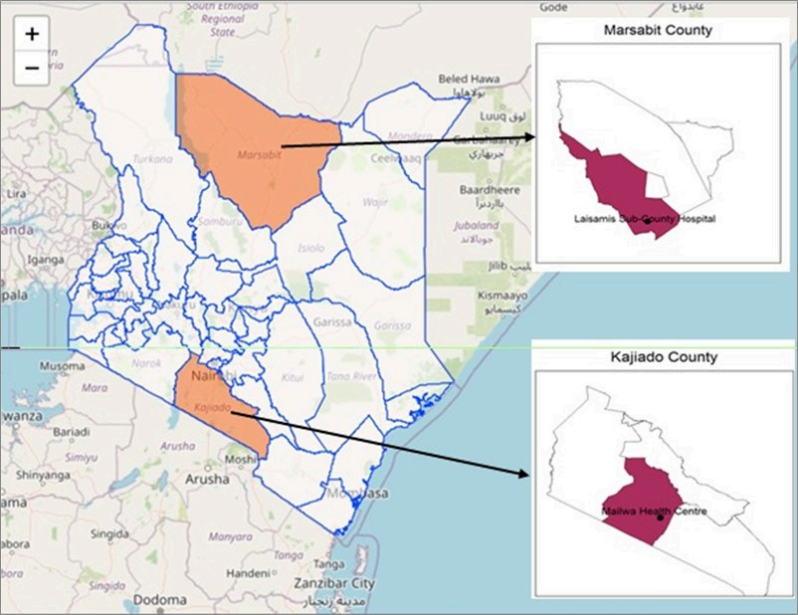
Map of East Africa Showing Marsabit and Kajiado counties and the study sites.

**Marsabit County**, is the largest, most arid and sparsely populated county in Kenya, covering at least 15% of the country’s entire territory. It shares a long running border with the Republic of Ethiopia to the North and regionally borders Wajir, Isiolo, Samburu and Turkana counties (Fig. 1). It covers a total area of 70,944 km^2^, and has a population of 459, 785 individuals belonging to 79, 273 households each with an average size of 5.8, and a population density of 6.481 people per km^2^ (KNBS, 2019). The county is inhabited by various communities including the Rendille, Borana, Gabbra, Somali, Sakuye, Wayyu, Burji, Samburu, Sitama, Konso, Daasanach and El Molo communities. The communities celebrate their traditions and diverse cultural heritage through language, attire, native recipes, dances, folklore, folk songs, artifacts and craftmanship (KNBS, 2015). Eighty percent (80%) of the inhabitants rely on pastoralism for income and livelihood. The majority keep mixed herds of camels, cattle, sheep and goats both for social and economic value, providing a critical context for zoonotic disease transmission. The main sources of water for human and livestock consumption are boreholes, shallow wells, springs, water pans and water holes found on dry riverbeds of the numerous seasonal rivers *(laga).* Administratively, the county is divided into four sub-counties namely: Moyale, North Horr, Saku and Laisamis. The study was conducted in the largely pastoralist subcounty of Laisamis, which is predominantly inhabited by the Rendille and Samburu communities.

#### Kajiado County

Situated in the southern part of Kenya within the Great Rift Valley, Kajiado County extends from the Nairobi metropolitan area to the north and boarders the Republic of Tanzania to the South (Fig. 1). It occupies 21,902 km^2^, with a population of 1,268,261 people, a population density of 31 people per km^2^ and an average household size of 3.4 (KNBS, 2019). It is characterized by semi-arid rangelands, vast plains, valleys, volcanic hills and scarce vegetation in low altitude areas which increase with altitude and rain. The county is primarily inhabited by the Maasai community, whereas the regions adjacent to Nairobi metropolitan area are multiethnic and rely on business ownership for livelihood. 42% of the inhabitants are pastoralists, 35% are in formal employment or casual labour, 12% are agro-pastoralists and 8% derive their livelihood from mixed farming (KNBS, 2020). The study was conducted within Mailwa and Sere villages of the Kajiado Central sub-county. About 75% of households within Mailwa engage in pastoralism as the main economic activity, predominantly rearing cattle, goats, and sheep.

### Participant recruitment, data collection and management

The study was conducted among livestock-rearing households residing within the catchment areas of Laisamis Subcounty Referral Hospital and Mailwa Health Centre. We employed probability proportional to size (PPS) sampling to select eligible households in 40 manyattas from seven villages in Marsabit and 71 manyattas from eight villages in Kajiado counties, respectively. A household was eligible if they reared at least one livestock species (cattle, camels, goats or sheep). For each village, the number of households selected was distributed proportionally based on the relative human and livestock population and total number of households in that village. In both sites, households are clustered into compounds or dwelling units called manyattas. Within each selected manyatta/cluster, systematic random sampling was applied as part of a prospective human-animal cohort study to evaluate the role of camels and other livestock species in the transmission of Brucella spp. and Middle East Respiratory Syndrome Coronavirus. Potential participants from enrolled households as well as key informants from the study catchment were eligible to participate in the qualitative component of the study and included elders, women, *morans* and community own resource persons such as ethno-veterinarians, traditional healers, local administrators, retired teachers, retired public servants and religious leaders. Once a potential key informant, focus group or individual interview participant was identified, the research assistants obtained written informed consent or assent plus parental permission – where an eligible participant was below the age of 18 years, before any study procedures could be undertaken. This included consent for audio recording as well as videography and photography whenever required.

**Figure 2:**
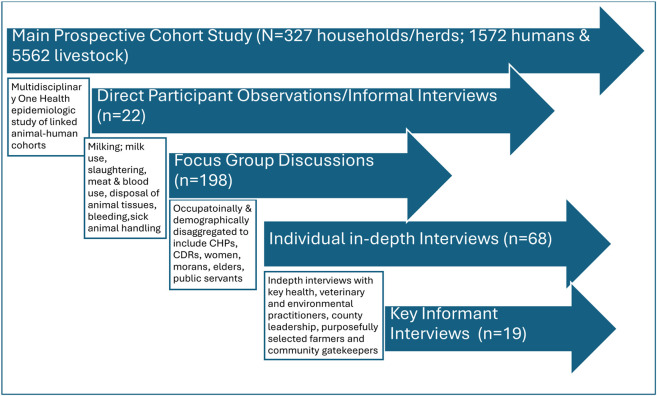
Schematic presentation and justification of the Qualitative data collection approaches. CHP-Community Health Promoters, CDRs-Community Disease Reporters

Qualitative data collection started with informal conversations and observation of pertinent participant practices. These multiple observations continued throughout the duration of the ethnographic enquiry and involved lay animal-parturition assistance, disposal of placentae and aborted materials, handling of dead animals, abattoirs & slaughter slab environment as well as pre-and post-mortem practices. This was followed by focus group discussions, individual in-depth interviews and finally with the key informant interviews respectively (Fig. 2). Informal conversations and go-along interviews were unstructured and employed necessarily to identify salient themes and patterns emerging from the data, including the multidimensional aspects of participant’s behaviors and cultural practices, movements, human-herd and her-herd interactions, embodied or situated knowledge and vulnerabilities in the most natural social context as well as appreciate shifts in power dynamics, daily routines and changes in their shared environment. They were captured through descriptive notes and occurred mostly in the *laga, fora* and *manyattas*. Unfocussed observations were captured through jottings which were soon-after expanded into field notes using thick descriptions. Focussed observation of relevant events, surrounding environments and spaces were captured using still photography. Individual in-depth interviews (n = 68) and focus group discussions (n = 20), were conducted in the participant’s preferred language using semi-structured guides designed specifically for this study. Each interview lasted approximately 60–90 minutes. The individual interviews were conducted within the *manyatta* or household dwelling while focus group discussions typically occurred at a central location outside the manyatta or communal meeting spaces where gatherings of similar nature are usually held. Each focus group comprised of 6–7 participants aggregated by age and gender. The age groups were 13–17-year-old, 18–29-year-old, 30–49-year-old and those aged 50 years and above.

Interviews with key informants (n = 19) involved understanding knowledge and practical gaps in community competence in prevention and control of brucellosis i.e. risk factors, risk perceptions, diagnosis, and treatment, including alternative therapy. The interview guide also included normative questions which solicited recommendations on practical and culturally appropriate interventions for prevention and control of livestock-borne zoonotic diseases such as brucellosis.

Initial discussions conducted between July and November 2023 included questions around animal husbandry, diversity of livestock diseases, knowledge of brucellosis, beliefs and practices on brucellosis, and health care seeking. The weather conditions during this period were generally wet following prolonged heavy rains during May-June 2023 period. Follow-up interviews and focus groups held in February 2024 focused on disease characterization, risk factors and health-seeking behavior and practices. The qualitative research team included a senior research fellow and two postgraduate students from the Department of Anthropology, Gender and African Studies at the University of Nairobi, which ensured adequate experience and quality-assured ethnographic enquiry.

All focus group discussions (FGDs) were digitally recorded and professionally transcribed and translated. Transcripts were reviewed for accuracy, and any personal identifying information was removed. All the original files were named by study site and informant source as part of the initial de-identification of the files. Subsequently, source-matching and data cleaning was done for all files to ensure all files correspond to the right participants and study sites. Language translation was done for files that were transcribed in Kiswahili to facilitate ease of coding. Local names and references to medicinal plants and animal diseases have been maintained for emphasis purposes and meaning-based English translations are included in Table 1. Data were stored in password-protected databases in the Washington State University secure server with access restricted only to authorized staff.

### Data analysis

We used NVivo 14 (version 14.23.2, Lumivero) to code and analyze the data. All data were analysed inductively using grounded theory where the process of data collection and theory development were iterative with the emerging theories and themes informing data analysis (Brink et al., 2006). Research findings were then integrated and presented as narrative descriptions and verbatim quotations.

The following steps were employed in data coding and ensuing qualitative analysis: (1). Data familiarization through reading and re-reading of transcripts to broadly understand participant answers and thoughts on each question; (2). Generating initial coding schemes by identifying recurring themes in the transcripts; (3). Generating initial themes by grouping related codes; (4). Discussing the coding scheme amongst the research team members, revising and integrating emerging themes from ongoing interviews to generate final themes. The step also included consensus-building and inter-coder reliability checks; (5). Naming themes; (6). Synthesizing primary findings while ensuring that our interpretations preserve the participant’s intent and (7). Reporting primary findings.

Four main themes emerged from the focus groups, individual in-depth interviews and informal conversations, namely: (1). Slaughtering, and meat consumption practices, (2). Milking and milk-use practices, (3). Assisted parturition and handling of placenta and abortion materials, (4). Zoonotic disease risk perception.

#### Ethical approval, consenting and participation

The study protocol was reviewed and approved by the Kenya Medical Research Institute’s Scientific and Ethics Review Unit (KEMRI-SERU reference # 4405), the KEMRI Animal Care and Use Committee (reference # KEMRI/ACUC/02.07.2022), and the Washington State University Institutional Animal Care and Use Committee (reference # ASAF 7081). Subsequently, the National Commission for Science, Technology and Innovation provided the research permit (NACOSTI/P/22/17621). All participants provided written informed consent before study participation.

## Results

We present data from 214 purposively sampled participants and 22 informal observations from Kajiado and Marsabit counties. Overall, 53.7% of the participants were male while 46.3% were female. Furthermore, 63.2% of the key informants, 55.9% of in-depth interviews participants and 51.2% of the focus group discussion participants respectively, were male as shown in Table 2.

### Slaughtering and meat consumption practices

Slaughtering is the act of killing an animal for food. It is done in designated places such as the municipality slaughterhouses or a selected place within the market yard or a random location within the homestead. In the smaller towns and municipalities without public health-sanctioned slaughterhouses, individual farmers and butchers set up their temporary slaughtering pens. The absence of protective equipment and hygiene measures throughout the process as well as potential environmental contamination through blood, offal and skin disposal was observed. The meat could also be contaminated by being in contact with a contaminated environment.

Participants reported that animal slaughtering was frequent for household consumption and for sale in the local butcheries. Slaughtering for sale happened daily while that for local consumption was mainly associated with specific cultural activities such as weddings, engagements, thanksgiving, funeral ceremonies, harvests, sacrifices, rites of passage or to celebrate specific occasions and achievements.

"I am a butcher and a slaughter. I slaughter animals and bring them to my butchery, and I also slaughter for the others to take to their butcheries. I have done this work for twenty-eight years. I also train local slaughterers."

(Male, IDI, Marsabit)

In both communities, the participants reported traditional ways of diagnosing livestock and human illnesses and used local references for them. This cultural competence or lay knowledge was acquired through apprenticeship and passed down generations. During slaughtering of any animal with suspected illness, they would particularly inspect the kidneys, liver and the heart first and tell what the animal died of. The participants also reported a common practice in both communities where the carcass of an animal that dies of suspected disease, is not discarded wholesome, but the farmer or butcher would carefully dissect out the parts that were presumably diseased and consume the rest. As a precautionary measure, the carcass from a dead animal or one slaughtered for being sick is handled cautiously to reduce the chances of afflicting the consumers.

"When a cow dies from that disease. There is a way we know, The Maasai call it ‘Imboruo’, when the cattle are slaughtered after five minutes the meat turns black. When it turns black it shouldn’t be eaten. If the meat is suspected to have been of cattle that was sick and has shown no sign, it must be thoroughly boiled before it is eaten."

(Female, IDI, Kajiado)

"When a goat is sick and is slaughtered, the disease can be identified by looking at the internal organs like the liver and heart. These are disposed of while the rest of the meat can be eaten."

(Male, IDI, Kajiado).

"We don’t eat camels these days because we don’t know what might have killed them. But for the other animals we boil the meat twice to kill germs then eat."

(Female, IDI, Marsabit)

Some participants in both communities engaged in dietary practices such as consumption of raw meat and fresh blood, including meat from wild animals, due to perceived nutritional benefit which is reportedly lost when the meat is cooked. Health professionals were equally oblivious to the potential risk of zoonotic disease exposure. They were initiated into raw meat and blood consumption as young *morans*, long before they became healthcare workers. One male animal health professional remarked:

"Of course, I am a trained expert and do meat inspection. So, I'll inspect it before eating but I do eat raw kidneys…I specifically do raw kidneys. I can also eat parts of the reticulum while raw. I also take raw blood. I don't do it in the butcheries where you have no idea how it is slaughtered."

(Male, KII, Kajiado)

"When cattle are slaughtered, the morans drink the blood immediately. Sometimes they drink it directly from the animal’s skin during slaughtering or bleeding."

(Male, IDI, Marsabit)

### Milking and milk utilization practices

**Figure 3:**
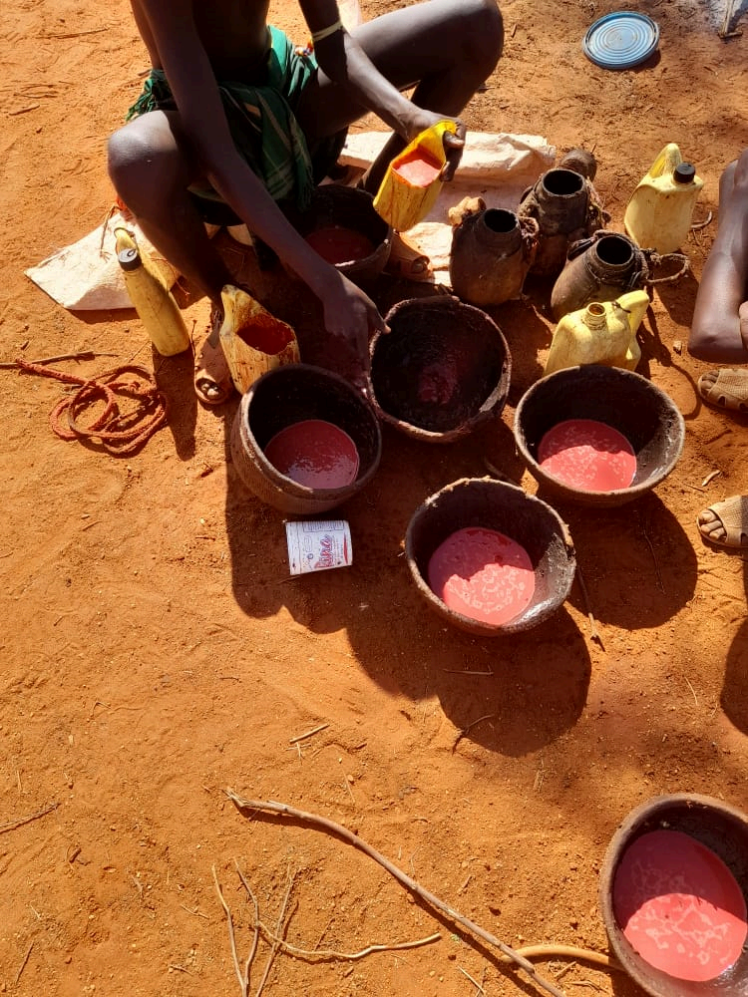
Photo of broth made from milk and blood (Sakalinja) and preferred by morans, Laisamis fora, Marsabit. Photo Credit: Sylvester Leruk.

**Figure 4:**
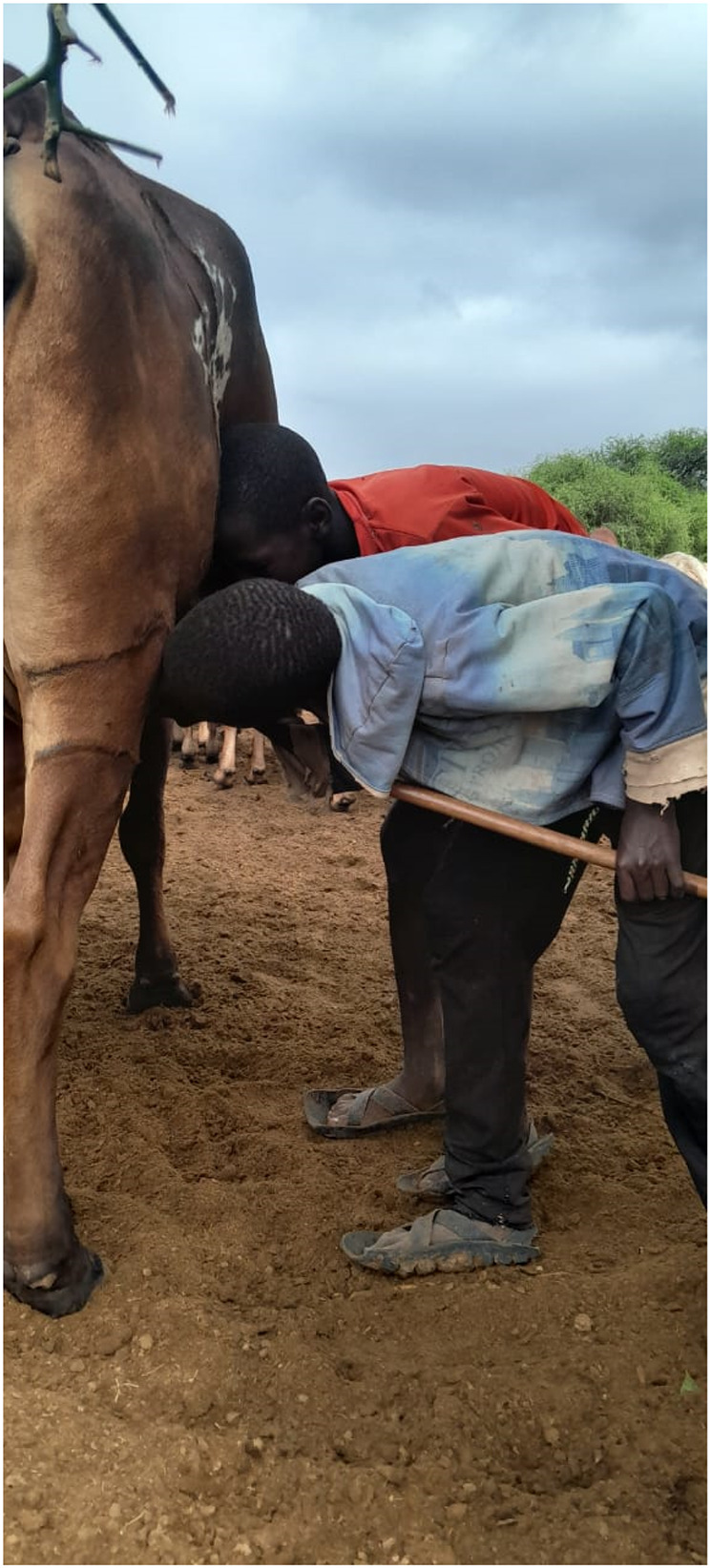
Photo of young morans drinking milk directly from lactating cow in Mailwa, Kajiado County. Photo credit: Raphael Pasha.

Many participants observed that milk was consumed according to the prevailing structures within the household, with children being prioritized. In both communities, milk is predominantly consumed fresh. It can also be fermented or mixed with tiny pieces of meat and blood to form a special meal referred to as *‘sakalinja’* (Fig. 3), which is preferred by the *morans* and herders because it sustains and keeps one full for prolonged periods, a highly desired food attribute under nomadic pastoralist lifestyles. Some participants do not boil the milk, citing various reasons including losing its taste or retorting that it is culturally prohibited. Young children and the elderly frequently drank unboiled milk while the young and uncircumcised boys would drink directly from the animal (Fig. 4).

"Cultural norms dictate milk handling, with beliefs against boiling milk and preference for raw milk consumption…you find people just milking and drinking it directly because they find it to be more nutritious and it has more vitamins, so they believe that once you boil milk, you have destroyed all the nutrients."

(Male, KII, Marsabit)

"When many cattle have given birth and the milk is in abundance, people forget to boil the milk. They just drink it immediately after milking. Even me…and sometimes you have a lot of work, and don't find the time to boil milk."

(Female, IDI, Kajiado)

#### Social structures and gender norms

Slaughtering, milking, and milk use practices in both communities were handled differently by age and sex. Participants reported that slaughtering was mostly performed by adult men and the *morans* while milking was mostly the work of women and children except camels and some cattle that were milked by adult men and *morans* only. Fresh milk was mostly consumed by children and elderly men, particularly where prioritization was required due to food scarcity. In both communities, the participants reported that women and children were at a greater risk of transmission of brucellosis in comparison to men:

"Milking is mostly done by women. In exceptional cases like when we have moved away from home in search of pastures, men do it because there are no women with them. It is mainly a woman’s job to milk."

(Male, IDI, Kajiado)

"I milk goats and cows, but camels must be milked by a man; it's just a tradition. Slaughtering and butchering animals are tasks for men, and on my part, cooking is my responsibility."

(Female, IDI, Marsabit)

### Assisted parturition

All animal deliveries are managed expectantly in both communities. When an animal is ready to deliver, it is often moved back to the homestead where it will be easy for delivery to be monitored. We observed and documented traditional practices associated with normal deliveries, abortions, and retained placentae. Frequently, assisted parturition occurred without the use of infection control measures or any protective gear, including by trained veterinary and para-veterinary professionals. Often, the nearest person present helps the animal to deliver.

"When a goat delivers, you help it and carry the kid home. Even the cattle we can help but mostly we help goats and sheep. After delivery, you carefully remove the placenta or leave it to come out on its own. If it gives birth there, I don’t use gloves, I use bare hands to help and carry the offspring home then I wash my hands. At the grazing fields, there is no water."

(FGD, Women->50 years old, Kajiado)

"We just help them without gloves, for someone like me who is learned after helping them I clean my hands, but then those from the remote villages don’t wash their hands because they think the amniotic fluid has no problem, so they just wipe their hands on the bark of trees."

(Male, IDI, Marsabit)

### Handling of placenta and aborted fetuses/materials

**Figure 5:**
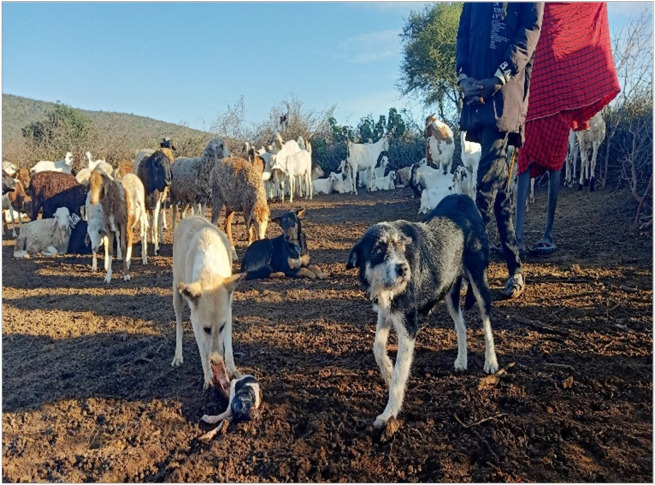
Photo of dogs feeding on aborted sheep fetus in Kurket village, Kajiado County. Photo Credit: Raphael Pasha.

Participants reported that the afterbirth or aborted materials are either left unattended or fed to the dogs. Other participants buried it or left it to rot. Nearly every household keeps at least one dog that often accompanies the herders to the grazing fields. The dogs scavenge on any food materials they come across in the field including carcasses of dead animals, placentae, and aborted fetuses (Fig. 5). Some farmers used sticks to pick up the placenta or aborted fetus while others used their bare hands.

"Yes, sometimes they give birth in the wilderness, and we just leave them there as long as the animal is free of the placenta. When not free you help pull it out."

(Men, FGD_18-29-year-olds, Kajiado)

"Placenta is left for dogs to eat if within the homestead, and for wild animals if outside. In case of miscarriage, the fetus is given to the dog…Sometimes 50 or more goats can miscarry in one season")

(Male, IDI, Kajiado)

### Zoonotic disease risk perception

Participants in both communities demonstrated knowledge of the various signs and symptoms of common zoonotic diseases in animals including abortions, delivery of weak offspring, retained placenta and reduced milk production. However, they attributed these signs and symptoms to various biophysical and cultural phenomena such as the use of herbal preparations and harsh weather. Some participants attributed abortions in livestock to sickness, the presence of tsetse flies, Q-fever, RVF and brucella while others reported drought-related causes such as lack of rain/water and pasture as well as use of some herbal shrubs. Some farmers do not believe reduced milk yield can be attributed to zoonotic diseases such as brucellosis.

"There are a lot of things that make goats abort, there is that disease called ‘lkipei’ that makes them abort or give birth to weak offspring, there is also the disease that makes them have wounds all over their bodies."

(Male, IDI, Marsabit)

"There are also those who believe that the cattle and the camel when dead should not be consumed but they want to recover the skin, so they skin the animal for their use and sale and bury the rest of the body and in the event become exposed which can lead to them coming down with zoonotic diseases, especially anthrax as it is spread by contact."

(Male, KII, Marsabit)

Some participants do not believe that zoonotic diseases such as brucellosis are transmitted through the consumption of contaminated. The use of fresh animal blood for anaemic patients is commonly practiced in both communities. Any animal slaughtered as part of therapeutics for a sick household member or as an antidote for snake bites or for circumcision rituals, the meat and blood are to be consumed fresh, raw and warm. Many participants cited various reasons for their beliefs; for instance, that they have always consumed milk like that for ages without any afflictions.

"We don’t know much about that (Brucellosis’). But what I know is that it is not caused by milk because it affects people during droughts where there is unavailability of milk. As Maasai we been keeping livestock for a long time, but we have never been affected by milk from our livestock therefore we don’t believe that Brucellosis is caused by milk."

(Male, IDI, Kajiado)

### Human-animal and animal-animal interactions

In both communities, livestock lived in proximity to the pastoralists. Young ones lived within the same house or a specially made unit but away from the older animals. There were pools of animal manure made up of animal excreta, (feces and urine) as well as secretions from the nose, throat, blood, vagina, mammary glands, skin, and placenta. The animals’ manure in some cases was not regularly disposed of, hence accumulating into multiple layers, leading to the potential of disease exposure from fomites, saprophytes, zoonotic microbes and soil-borne helminths as well as posing an additional risk of physical injuries to the farmer – from accidental falls, during routine caring for the animals. Furthermore, increased chances of insect and rodent vectors from the unmanaged pools of animal waste could increase the potential for vector-borne disease exposure.

Free mixing and comingling of different animals and herds occurs frequently in the grazing fields, *laga*, and in livestock markets in both communities. Furthermore, there is a common cultural practice in both communities where farmers lend their breeder bulls to their neighbors and friends for breeding purposes. Lactating animals are also frequently lent to less privileged communities/family members for food security.

"You can’t tell someone that you need his breed, but you will just ask for a bull to mate with your cows. Sometimes they might not be able to give it to you instead, you take your cows to his farm."

(Male, IDI, Kajiado)

"During grazing they mix, and they mate with different cows and there is no problem with that."

(Male, IDI, Kajiado)

## Discussion

Zoonotic diseases such as brucellosis remain prevalent among pastoralist communities in low and middle-income countries where they pose enormous public health risks and adverse socioeconomic impacts. Our study explored the cultural and behavioral factors that influence the transmission, endemicity, and control of zoonotic diseases among select pastoralist communities in East Africa with the overarching goal of understanding how these insights can inform more effective public health interventions. The study focused on brucellosis and utilized ethnographic fieldwork, in-depth interviews, focus groups, and participant observation to explore and gather comprehensive data on the various cultural, social, and environmental factors influencing zoonotic disease transmission and control among pastoralist communities.

Consumption of raw milk and home-based slaughtering practices are frequent in these pastoralist communities. Home-based slaughtering was performed without adequate hygiene measures. It was mostly done by adult men and the *morans* hence increasing their risk of livestock-borne zoonotic disease transmission disproportionately. The slaughter slabs and medium slaughterhouses frequently operated without strict adherence to the provisions of the Meat Control Act and other public health protocols for slaughtering and distribution of meat for human consumption (National Council for Law Reporting, 2012). The local butcheries lacked slaughterhouse licenses from the Director of Veterinary Services. After slaughtering, the meat was often not inspected by a trained public health officer. These findings are similar to observations by Owiny et al., (2023) where more than half (57%) of participants identified consumption of uninspected meat as the main risk for infection with zoonotic diseases among communities living at the human-livestock-wildlife interface.

Dietary practices such as consuming raw meat and blood, including meat from wild animals, were commonly practiced by in both communities. These practices were attributed to the local culture and long-standing traditions. For instance, Oiye *et al* (2006) found that the Maasai pastoralists rely on meat, milk and blood from cattle for protein and energy needs. In this community, fresh animal blood was consumed whenever an animal was slaughtered or when a household member lost substantial amounts of blood during childbirth or circumcision to replenish their low blood levels (ibid). From a scientific lens, consumption of blood does not provide nutritional benefit, since humans did not evolve the metabolic capacity to digest and assimilate blood (Katherine Ramsland, 2002). Instead, such practices could carry severe health risks, including the risk of contracting bloodborne illnesses or food poisoning, especially if the blood is not collected hygienically. Where an animal was slaughtered for therapeutic use, as an antidote for snake bites or circumcision rituals, the meat and blood were consumed fresh, raw and warm. It is believed that the nutrients are intact when products are consumed raw as boiling or cooking destroys the nutrients.

Consuming unpasteurized milk can pose a significant risk of infection with milk-borne zoonotic pathogens such as Brucella and *Mycobacterium bovis*, which may be present in the milk of infected animals. For instance,Leone et al., (2022) demonstrated the presence of microbiological, chemical, and physical hazards in milk and milk products, whileDavis et al., (2014) highlighted several health risks associated with consumption of raw milk, emphasizing the need for improved hygiene practices. Additionally,Muunda et al., (2023) found that 98% of households in peri-urban and low-income settings in Kenya purchased unprocessed fresh milk at least once during the 7 days before the survey, while only 17% purchased packed pasteurized milk. Furthermore, if a particular household doesn’t boil milk or practice other food hygiene measures, everyone in that household will end up taking raw milk. This is a structural problem requiring intervention at the household level.

Pastoralists often assist in the birthing process without using protective equipment, which increases the risk of transmission through contact with birth fluids and tissues. This lack of protection is attributed to low perception of zoonotic risk as well as lack of necessary appropriate personal protective equipment that are easily accessible to farmers. Many deliveries requiring some assistance to the parturient and neonate were performed by adult men or *morans*, hence increasing their exposure to diseases disproportionately.Zinsstag et al., (2011) emphasize that the lack of protective measures during such high-risk activities contributes significantly to the spread of zoonotic diseases, including brucellosis. Further, they posit that livestock diseases that cause abortion may inherently also cause retention of the placenta and other birth complications.

Handling of placentae and aborted materials/fetuses carries significant risk for the transmission of zoonotic pathogens including *Brucella spp.* since these materials and secretions can be heavily contaminated with microbes that shed predominantly in reproductive tissues González-Espinoza et al., (2021). Unsafe handling of aborted materials and placentae observed in our study was similar to the findings from an empirical One Health study on zoonotic risks from livestock birth products among rural communities in Ethiopia Alemayehu et al., (2021), which found substantial knowledge gaps, a low level of the desired attitude, and high-risk behavioral practices regarding zoonotic disease from livestock birth products. The risk of environmental contamination with abandoned infectious afterbirths can increase the transmission risk of zoonotic pathogens such as *Brucellae* that can survive in the environment for prolonged periods of up to three months thus exposing several generations of livestock and wildlife that encounter a single contaminated field Abubakar et al., (2011).

During seasonal migrations, in search of water and pasture, pastoralists may graze their livestock in areas with an increased likelihood of encountering other livestock herds, infected wildlife and wildlife reservoirs of diseases like anthrax and brucellosis. Several studies evaluating livestock movement in pastoralist communities have established the important role mobility of pastoralist herds play in the spread and control of zoonotic pathogens Ekwem et al., (2021); Klous et al., (2016); Omondi et al., (2021). Communal water sources often shared by pastoralists can become contaminated with zoonotic pathogens which can subsequently spread among herds and from animals to humans.

Perception of zoonotic disease transmission risks among pastoralists is deeply rooted in their traditional beliefs and cultural practices, which can affect the acceptance of scientific explanations and modern interventions. A study by Grace et al., (2012) highlights how local perceptions of zoonotic diseases influence the adoption of preventive measures in African communities. In the present study, some participants demonstrated knowledge of the various signs and symptoms of common zoonotic diseases in animals including abortions, delivery of weak offspring, retained placenta and reduced milk production. However, they attributed the signs and symptoms to various biophysical and cultural phenomena including the use of herbal preparations, harsh weather, negligence by health workers and non-specific livestock illnesses. This belief system can hinder the recognition of the risks associated with zoonotic transmission. Another study by Grace et al., (2008) found that pastoralists often did not associate handling of animal reproductive materials with disease transmission leading to a lack of precautionary measures which can threaten zoonotic disease prevention and control efforts.

Both genders recognize and adhere studiously to their culturally prescribed roles. For examples participants in women focus groups recognized that milking and preparation of meals was their role while their men performed roles such as slaughtering. This is similar to findings from previous studies in pastoral communities where gender roles are deeply rooted in sociocultural mores that limit women’s ownership and care of livestock to small stock while camels and cattle are mostly owned and managed by men, Fusco et al., (2022); Oruganti et al., (2023). Involvement of women and children in milking and caring for young animals, combined with their roles in food preparation and household resource management, places them at higher risk of exposure to zoonotic diseases Flintan, (2008); Kristjanson et al., (2010).

Overreliance on cultural competence and traditional remedies can delay the seeking of formal medical or veterinary care, potentially worsening the outcomes of zoonotic diseases. The ethnoveterinary diagnostic ability of the Maasai traditional healers cannot be overstated Ole-Miaron, (2002). While traditional healers play a crucial therapeutic role in the community, their involvement in disease control efforts can be beneficial in the short term but detrimental eventually, particularly with chronic illnesses where delay in seeking medical care can lead to long-term complications. Since traditional healers are trusted figures within the community, engaging with them alongside other community leaders in educational campaigns for prevention and control of zoonotic diseases can help bridge the gap between traditional beliefs and modern scientific understanding of disease transmission and mitigation measures, as previously observed by Kamuya et al., (2013). A review of systematic reviews on community engagement interventions for communicable diseases in low- and middle-income countries demonstrated that the extent of population coverage, shared leadership and community control over outcomes were the most important factors influencing the effectiveness of community interventions Questa et al., (2020).

Effective control of zoonotic diseases in pastoralist communities requires a multifaceted One Health approach that addresses the cultural, behavioral, and socioeconomic factors influencing disease transmission and endemicity. In the absence of effective biomedical interventions such as mass livestock vaccination, educational initiatives that are culturally sensitive, tailored to the local context of pastoralists and incorporate local stakeholders, beliefs and practices can significantly enhance understanding and utilization of public health interventions for prevention and control of zoonotic diseases.

### Study limitations

We recruited participants from two pastoralist communities, the Maasai and Rendile communities of Kajidao and Marsabit counties, respectively. Hence, our findings may not be transferrable to other communities in different pastoralist settings. Another limitation of this study is the inherent potential for cultural and language barriers which could introduce bias in accurately interpreting and contextualizing the information gathered; especially in regions where nuanced cultural practices related to livestock handling, slaughtering, and milking can vary significantly. Misinterpretations or oversimplifications of these practices could result in an incomplete or skewed representation of the factors influencing disease transmission and control.

Given the similarity of the cultural beliefs and practices among pastoral communities in geographically diverse regions, we believe our findings provide valuable insights into zoonotic disease risk perceptions and practices that are applicable in many cultural contexts and pastoralist settings in low- and middle-income settings.

## Conclusion

Effective control of zoonotic diseases in pastoralist communities requires a multifaceted One Health approach that addresses the cultural, behavioral, socioeconomic and environmental determinants of disease transmission and endemicity. This study explored the social, structural, cultural and behavioral factors that influence the transmission, endemicity, and control of zoonotic diseases in pastoral communities, with a particular focus on understanding how these factors can inform more effective public health interventions.

Traditional knowledge systems and local beliefs play a crucial role in shaping community responses to disease outbreaks and control measures. Persuading communities to adopt practices such as boiling milk or using protective equipment during animal handling requires not only education but also cultural sensitivity and meaningful engagement with community leaders. Knowledge and beliefs about zoonotic diseases vary widely among pastoralist communities; while some communities have a high awareness of the symptoms and transmission routes of diseases like brucellosis, others may have limited understanding and/or misconceptions.

We recommend development and implementation of culturally tailored interventions that integrate traditional knowledge and cultural competence of pastoral communities with modern veterinary and medical practices to create culturally acceptable zoonotic disease control measures. Sensitization of the local community leaders and members about the burden, risk factors, transmission dynamics and prevention efforts of common zoonotic diseases is critical to bridge the knowledge and sanitary practice gap in a culturally sensitive manner.

## Figures and Tables

**Table 1:** Glossary of Maasai and Rendile indigenous names and local terminologies

Indigenous Name	Root	Meaning/Application
*Manyatta*	Maasai, Samburu and Rendile	A traditional hut made from sticks and grass or other locally available material, used by the Maasai and Samburu communities in East Africa. Also refers to a group of huts or compounds established within a common fence by a family or clan.
*Laga*	Maasai, Samburu and Rendile	A man-made watering point or shallow well usually within the track or base of a seasonal river where pastoralists, their families and livestock access water. Also refers to the track/bed of a seasonal river.
*Morans*	Maasai, Samburu and Rendile	Young warriors, traditionally between adolescence and adulthood, whose primary role is to protect the community and their livestock. The young men are initiated through rite of passage during which they live in isolation, often in the bush, learning their culture and customs as well as developing strength, character, courage, and endurance.
*Fora*	Maasai, Samburu and Rendile	Temporary satellite nomadic camp/ dwelling unit established by pastoralists usually in distant grazing lands away from the farmer’s permanent home/village, used during drought to reduce the distance between searching pasture and water for livestock and human consumption.
*Imboruo*	Maasai	Black quarter disease. A bacterial infection of young cattle, sheep, and goats caused by *Clostridium spp.* It characterized by emphysematous swelling in the hind limbs, severe toxemia, and high mortality rate.
*Sakalinja*	Maasai, Samburu and Rendile	Special meal made of fresh blood mixed with milk and tiny pieces of meat.
*Lkipei*	Maasai, Samburu and Rendile	Contagious caprine pleuropneumonia/pneumonia. A serious bacterial disease affecting goats caused by *Mycoplasma capricolum* and characterized by severe respiratory distress, nasal discharge, coughing, dyspnea, pyrexia, pleurodynia, and general malaise.

**Table 2:** Number of participants by data collection method in Marsabit and Kajiado Counties.

	Marsabit County			Kajiado County			Total Both Counties		
Data Collection Method	Male	Female	Total	Male	Female	Total	Male	Female	Total
FGDs			10			10			20
FGD Participants	31	29	60	34	33	67	65 (51.2%)	62 (48.8%)	127
IDIs	20	18	38	18	12	30	38 (55.9%)	30 (44.1%)	68
KIIs	8	3	11	4	4	8	12 (63.2%)	7 (36.8%)	19
Total participants							115 (53.7%)	99 (46.3%)	214
Informal observations&conversations	Slaughtering (in the abattoir and in the local market), milking (of both large and small ruminants), living arrangements (within household and in manyatta), *laga* (interaction of herds at watering points), *fora* (human-herd and herd-herd interactions, dietary practices), dietary habits, sanitary practices						22		

FGD = focus group discussions, IDI = individual in depth interviews, KII = key informant interviews,.

## Highlights

- Most pastoralists know and refer to common zoonotic diseases by their local names.
- Therapeutic or ritual consumption of raw animal products increases zoonotic risk.
- Women and children face higher zoonotic risk exposure from milking and animal care.
- Behavior change communication with cultural relevance aids zoonotic control.
- Local leaders pivot health interventions through culturally sensitive engagement.

## Abbreviations

DTRA: Defense Threat Reduction Agency
FGD: Focus Group Discussion
IDI: Individual In-depth Interview
IPC: Infection Prevention and Control
KEMRI: Kenya Medical Research Institute
KII: Key Informant Interview
KNBS: Kenya National Bureau of Statistics
RVF: Rift Valley Fever
Spp.: Species
WOAH: World Organization for Animal Health
